# 28 Substance Use Disorder: A National Survey

**DOI:** 10.1093/jbcr/irae036.028

**Published:** 2024-04-17

**Authors:** Van Dobbe, Curt C Bay, Karen J Richey, Kevin N Foster

**Affiliations:** A.T. Still University, Mesa, AZ; Arizona Burn Center Valleywise Health, Phoenix, AZ; A.T. Still University, Mesa, AZ; Arizona Burn Center Valleywise Health, Phoenix, AZ; A.T. Still University, Mesa, AZ; Arizona Burn Center Valleywise Health, Phoenix, AZ; A.T. Still University, Mesa, AZ; Arizona Burn Center Valleywise Health, Phoenix, AZ

## Abstract

**Introduction:**

Substance use disorder (SUD) involves an individual’s inability to control their use of drugs, alcohol and/or medications. Our burn center provides care to over 1,400 inpatients and 5,000 outpatients a year, and we see numerous patients with this disorder who have complex and challenging needs that can affect staff member perspectives on providing care. To establish a broader outlook on the relationship between burn center staff and patients with SUD, a survey was conducted within our center in 2022 and the same survey was sent to burn centers nationwide in 2023.

**Methods:**

A modified version of the 11-item Medical Condition Regard Scale was distributed via SurveyMonkey to burn professionals across the country. It was requested that they distribute to team members as appropriate. An additional 6 burn specific questions were asked. Participants were asked to respond on a 6-point Likert scale. There were both positively (POS) and negatively (NEG) phrased questions. No personal or professional demographic data was collected to ensure anonymity. Binary frequencies were calculated.

**Results:**

A total of 60 individuals responded. When responding to POS questions, the majority agreed that insurance coverage should be equal to other conditions (88%), would not mind being called in to care for this population (53%), felt they could find something to help the patient feel better (67%), and found working with SUD patients satisfying (50%). The majority did not enjoy giving extra time to burn patients with substance use disorder (60%). When asked NEG questions, 38% responded it was difficult to work with these patients, 25% reported irritation, 23% preferred not to work with SUD patients, 20% felt there was little they could do to help, and 7% felt it was a waste of medical dollars. When asked POS burn specific questions, 68.3% felt current practice to manage withdrawal symptoms was effective, 65% felt that these patients experienced more anxiety, had other mental health disorders (86%) and that pain management was particularly difficult (90%). When asked NEG burn specific questions 48% felt the care they provided was appreciated, and only 58% felt safe when providing care.

**Conclusions:**

Overall response rate was low. Similar to the 2022 survey, the national survey indicated that staff were willing to help these patients yet reported difficulty in working with this population. Likewise, nationwide survey responses identified management of pain and withdrawal symptoms as areas of care requiring improvement. Of interest in our original survey 65% of staff feel compassionate toward burn patients with substance use disorders while 2023 survey results indicate 43% of staff feeling compassionate toward burn patients with SUD.

**Applicability of Research to Practice:**

Survey results can be used to develop protocols and improve patient outcomes as well as patient and staff satisfaction.

